# Can Consumers Judge the Freshness of Fish from Visual Cues? A Case Study of Japanese Consumers

**DOI:** 10.3390/foods13193191

**Published:** 2024-10-08

**Authors:** Penglong Li, Yutaro Sakai, Hisashi Kurokura, Nobuyuki Yagi

**Affiliations:** Graduate School of Agricultural and Life Science, The University of Tokyo, Bunkyo, Tokyo 113-8657, Japan; lipenglong@g.ecc.u-tokyo.ac.jp (P.L.); a-sakai@g.ecc.u-tokyo.ac.jp (Y.S.); akrkrh@outlook.jp (H.K.)

**Keywords:** fish freshness, sensory features, consumer perception, principal component analysis, fish eye luminance, fish body color

## Abstract

In contemporary markets, fish are frequently wrapped in cling film, necessitating consumers’ reliance on visual cues to assess freshness. This study explores whether common Japanese consumers can accurately discern fish freshness based solely on visual information. We conducted an online experiment with 529 randomly selected participants in Japan by asking them to select the freshest fish from photographs of horse mackerel with varying freshness levels. In order to elucidate the mechanism of freshness detection, we conducted principal component analysis on the quantified body color and shape data. Additionally, we measured physical characteristics such as lipid contents, gloss, length, and weight of the fish. This study examines the correlation between these visual cues and consumers’ judgments, revealing the accuracy of visual indicators used by consumers in daily assessments of fish freshness. These findings suggest that a portion of Japanese consumers can correctly identify the freshness of fish based on appearance. They primarily rely on the brightness of the fish’s eyes and specific color combinations of the body to judge freshness, with the shape of the fish having less impact. Comparing the selection frequency between high- and low-accuracy participants, we observed that a low accuracy in freshness detection was not solely due to a lack of sensitivity to signals from photographs, but may also result from a misinterpretation of these signals by consumers.

## 1. Introduction

The freshness of the fish is critical. Fish freshness has been the primary criterion used by consumers to evaluate the quality of fish products. One study revealed that consumers prefer freshness when purchasing fish products, followed by price, quality, packaging, and nonseasonal availability [1]. Another study showed that consumers are willing to pay higher prices for fresh fish [2]. Furthermore, some consumers equate fish quality with freshness [3]. However, consumers in the modern era face barriers in assessing the freshness of fish in grocery stores due to recent changes in distribution and sales methods. Fish in grocery stores are tightly wrapped in plastic to prevent dehydration and contact with bare hands. Simultaneously, online shopping only reveals visual information in the photos indicating the origin and date [4]. In today’s system, consumers must often judge the freshness of fish using only visual information.

The goal of this study is to investigate whether and how consumers can accurately judge fish freshness based solely on visual information. We designed an online experiment in which 529 consumers were invited to select the freshest fish from photographs of horse mackerel with varying levels of freshness sampled from retail stores in Tokyo [5]. To understand the mechanism of freshness assessment in depth, we conducted principal component analysis on the quantified data of fish body color and contour shape, extracting ten principal components representing color characteristics and nine representing shape. We also measured the physical characteristics of the fish, including lipid content, gloss, length, and weight [6]. We explored the correlation between these visual cues and consumers’ judgments of fish freshness. Furthermore, we compared the differences between the high- and low-accuracy participants to identify the features they used to judge the freshness of fish.

There are various methods to determine freshness; however, they are usually complex and time-consuming. These include chemical analysis, sensory evaluation, and advanced technologies such as fish analyzer and electronic noses [7]. In terms of chemical analysis, [8] detailed several core methods, including the determination of the K-value, peroxide value, TVB value, and redox potential [9]. It is worth noting that these methods, while providing us with large amounts of chemical information, are often very time-consuming and can cause damage to the sample. Recently, simple devices called Fish Analyzers Pro, which assess the freshness and lipid content of fish quickly by measuring body impedance, became available for observing fish quality during inventory and distribution [10].

The most important innovation of this study is that it is the first to examine general consumers’ ability to judge freshness based on appearance. Nakamura et al. [11] demonstrated that professionals could judge freshness based on appearance (color). Jarmin et al. [12] and Rocculi et al. [13] found that RGB color changes in fish eyes and gills correlated with fish deterioration. Karoui et al. [14] explored the use of mid-infrared (MIR) spectroscopy combined with chemometric tools to determine fish freshness. Olafsdottir et al. [15] attempted to determine freshness using a multisensory approach. Hicks et al. [16] conducted an internet survey to assess consumers’ knowledge and attitudes toward seafood and seafood consumption. However, it is unclear whether the general consumer can determine the freshness of fish by appearance in the current distribution and marketing system. Secondly, we simultaneously included multiple types of visual information (i.e., color and shape) in our analysis, which allowed us to shed more light on the consumer freshness assessment process. Murakoshi et al. [17] focused on the relationship between eye luminance and freshness, while Costa et al. [18] and Ohta [19] examined the role of color calibration in assessments of fish freshness. Our study built upon this by examining a comprehensive list of characteristics. Furthermore, the study compared data from fish analyzers with consumer judgment [20]. This approach pioneered the study of how consumers perceive and interpret visual cues associated with scientifically measured freshness levels. By combining objective measurements from a fish analyzer pro with consumer assessments, we can delve into the nuances of consumer sensitivity to specific visual signals.

## 2. Materials and Methods

### 2.1. Animal Ethics and Confidentiality

This study did not involve live animal experimentation or any harmful treatment of fish. This study only involved horse mackerel that were sold and purchased from retail stores. The experimental procedures were non-invasive, consisting solely of photographing the fish and measuring parameters such as freshness, size, and weight. In addition, the online survey did not collect any information that may reveal the identity of the respondent, nor did it include any questions that are potentially harmful to the respondents. The school policy states that we do not need an Institutional Review Board’s approval in such studies.

### 2.2. Freshness Definition

Definitions of freshness in the literature may vary but are usually based on time, such as the time since capture or the time since the fish was delivered to the store. Although time-based definitions are convenient, they are not practical in reality because the condition of the fish can vary greatly depending on handling and storage methods [2,21] To simulate real situations that consumers face when buying fish, relying solely on the number of days since capture to judge freshness can lead to accurate judgments in experiments but errors in real-life situations. Therefore, in this analysis, it is preferable to assess fish freshness based on the condition of the fish at the time of sampling rather than the number of days since capture. For this purpose, we used a fish analyzer pro to measure the freshness of fish. The principle of the fish analyzer pro is based on impedance, which measures how difficult it is to pass an electric current through the fish. High-frequency currents pass through cells with low impedance, while low-frequency currents pass outside of cells with high impedance. As the freshness of the fish decreases, the impedance also decreases due to the intracellular fluids flowing out of the cell [22]. By comparing impedance at different frequencies, the freshness of the fish can be accurately measured.

Recent developments in impedance-based freshness measurement methods have increased its popularity, with studies by [23,24] demonstrating the effectiveness of the Fish Analyzer for various fish species. Yuan et al. [10] introduced the C-value (the ratio of impedance between 2 kHz and 100 kHz) and found a high correlation with K-values, suggesting that impedance measurements reliably reflect fish freshness. Additionally, Okabe and Murata [25] confirmed the practical application and effectiveness of the Fish Analyzer Pro in the market, showing that it provides reliable freshness measurements that align well with the perceptions of seafood sellers and buyers.

### 2.3. Experimental Design

To investigate whether consumers can accurately assess the freshness of fish based solely on visual information, we conducted an internet questionnaire survey using photographs of horse mackerel purchased from fish shops. The survey questions were created by authors, and an actual online survey was conducted by Cross Marketing Inc. A total of 529 participants were recruited randomly, including 265 males (50.1%) and 264 females (49.9%) ranging from 16 to 76 years old. The participants represented a diverse range of occupations and were from all 47 prefectures of Japan. The experiment was conducted as a multiple-choice questionnaire in which participants were shown three photographs of horse mackerel sampled from retail stores in Tokyo and asked to select which they perceived as the freshest one. We used 242 fish for the experiment, and all fish were purchased from different retail stores to ensure various freshness levels. These fish were a mixture of both aquaculture and wild-caught. These photographs were randomly selected and combined from a pool of 242 images of horse mackerel with varying freshness levels, with every image appearing to participants at least 52 times. Each participant was required to complete 10 such selection quizzes. Subsequently, we analyzed the correlation between the frequency of selection, color, and shape features of the fish and measured values (i.e., freshness, lipid contents, length, weight, and gloss) of the fish. The frequency of selection (*S*) is expressed as

(1) $$S=\frac{number\;of\;times\;selected\;by\;participants}{number\;of\;times\;shown\;as\;an\;alternative\;}$$

### 2.4. Extraction of Visual Information

**Figure 1:**
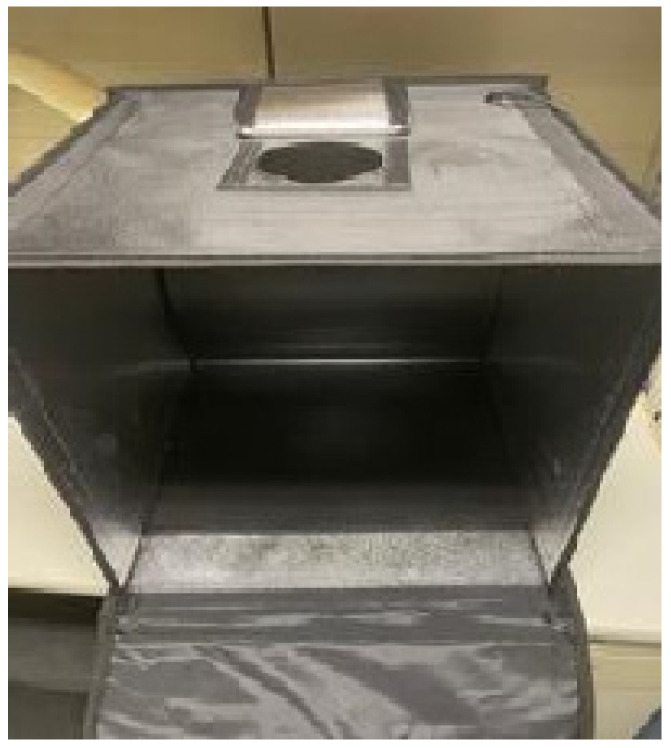
Shooting frame.

The gloss of the fish’s surface was measured within 10 min of purchasing the fish using a Gloss Measuring Instrument (Horiba IG 340). The fish were then photographed in a camera box (Figure 1). The freshness and lipid contents measurements were based on the electrical characteristics of the fish’s body, which were measured using a Fish Analyzer Pro DFA110 Ver.1.00 [5]. This can instantly measure the freshness and lipid contents of fresh fish without damaging its surface using impedance at five different electric current periodicities (General Scale Manufacturer, Yamato Scale Co., Ltd.: https://www.yamato-scale.co.jp/en/example/case/206 (accessed on 27 September 2024)). The selection of eye luminance and skin luster as visual cues are based on the knowledge obtained by the previous studies in assessing fish freshness. Cloudy white eyes and reduced skin gloss are commonly associated with poor freshness [17,26,27]. Therefore, these indicators were prioritized at our study in evaluating freshness.

The shooting frame was a square measuring 44 × 44 cm. Two rows of LED lights were placed on top of the frame, and the bottom center was marked to position the fish (Figure 1). The fish were placed in the center of the plate with the head facing left and the front facing up to ensure that the fish were in the same position and placed in the same way each time a photo was taken. The digital camera was fixed to the position of a hole to ensure identical shooting angles. The shooting box was fully enclosed and light sources were provided by LEDs on both sides to ensure adequate light and stability. The camera model used was a Canon PSD20 PowerShot D20 Digital Camera, approx. 12.1 Megapixels, 5× optical zoom, and Tough waterproof. After taking photos, the fish were stored on ice and brought back to the laboratory at the end of the day of purchase to measure their length and weight. After collecting 242 photographs, visual information was extracted from them.

**Figure 2:**
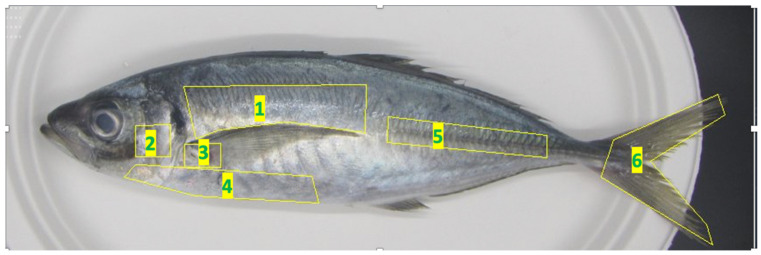
Six-part color analysis figure with the same formatting. Note: 1: Part 1 of the fish body; 2: Part 2 of the fish body; 3: Part 3 of the fish body; 4: Part 4 of the fish body; 5: Part 5 of the fish body; 6: Part 6 of the fish body.

Based on previous studies by [11,18] and observations of the photographs captured, we examined six distinct body parts, as depicted in Figure 2. Parts 1, 4, 5, and 6 were selected based on their significant color differences, whereas parts 2 and 3 were selected based on samples being easily damaged and reddish. The average color of each part was quantified using the Lab color space system, employing the OpenCV and Skimage libraries in Python. To assess the luminance of the fish’s eyes, we initially separated the eyes using ImageJ software (ImageJ2 2.9.0/1.53t) [28]. Subsequently, the average eye luminance was calculated with the “imager” package in R (Version 1.3.959), which is designed for image processing.

For analysis of morphological changes, a software tool called Shape (Ver. 1.3, an elliptical Fourier descriptor) was used to quantify the contour lines of the fish [29]. We binarized the photographs using “Paint 3D “to make shadowgraphs and converted the format of the photographs from JPG to BMP to adapt to Shape. In this process, special attention was paid to manually removing the body shadow caused by lightning. We put these shadowgraphs into the software “shape” for Fourier Transformation and principal component analysis. “Shape” extracts contours from shadow graphs of objects and abstracts these contours into the average amplitude of 20 frequencies using elliptical Fourier expansion. It then performs PCA on the variance and covariance matrix of these 20 frequencies using PrinComp in R (Version 1.3.959). As a result, “Shape” identifies 20 principal components and provides a scree plot of the eigenvalues (variance). It also generates contours for each principal component, showing the average amplitude of each frequency and contours representing the average ± 2 standard deviations.

### 2.5. Principal Component Analysis

To condense the body color data, we performed principal component analysis (PCA) using R software (Version 1.3.959). PCA is a dimensionality reduction technique that transforms the original variables into a smaller set of uncorrelated variables, called principal components while retaining as much variance as possible [30,31]. This approach is beneficial when dealing with complex datasets, such as color values across multiple body parts of the fish, by reducing the number of variables while maintaining the key information [32]. For the color information, we utilized the LAB color space, which is more intuitive for the human eye than RGB color space using three primary light colors. Each body part of the fish was characterized by three color values (L, a, b). In the Lab color space system, “L” represents luminance, with values between 0 and 100; “a” represents the hue from green to red (the negative axis represents green, and the positive axis represents red); and “b” represents the hue from blue to yellow (the negative axis represents blue, and the positive axis represents yellow) [33].

Given that we analyzed six body parts, this resulted in 18 color values per fish. These color data were then subjected to PCA to extract the principal components. Due to the complexity of the color data (comprising 18 variables) and the subtle variations in them, we adopted the cumulative variance contribution criterion to select the principal components. We chose the widely used standard of 80%. In the principal component analysis of the color data, the first 10 principal components met this standard. We explained the 10 color principal components by using the size of each principal component’s loadings, along with the color changes in the images corresponding to the variations in principal component scores. We examined the loadings of each color variable to interpret principal components. The loadings indicate how much each original color variable contributes to a principal component. By observing the visual changes in the images, the significance of the color in principal components can be explained. An explanation of each principal component is given in Table 1.

The software “shape” returned principal components with the result of the scree plot. We selected 9 principal components from them referring to the scree plot. The description of the morphological changes expressed by each principal component is given in Table 2.

### 2.6. Participant Grouping and Analysis of Variance

To explore how people with high accuracies differ from those with low accuracies when judging freshness based on fish appearance, we analyzed participants’ preferences in judging fish freshness by standardizing the characteristics of the fish selected by each participant. Participants were classified into three groups based on their correctness rates. To explore variations in freshness across these groups, we calculated their average scores and conducted a one-way analysis of variance (ANOVA) [34].

## 3. Results

### 3.1. Distribution of Correct Answers

**Figure 3:**
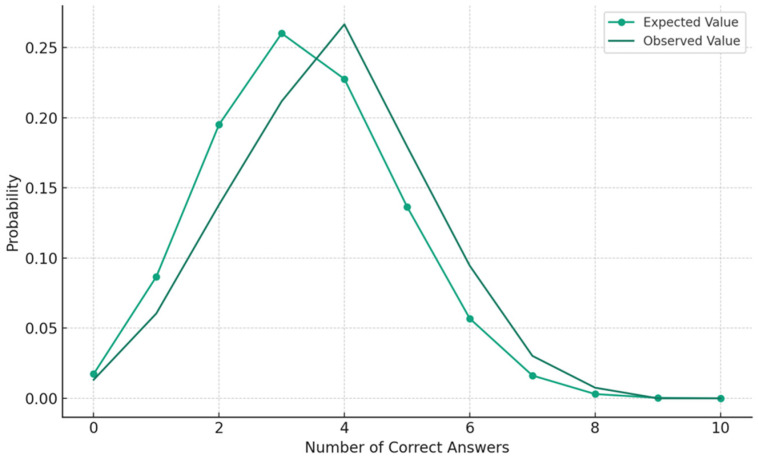
Distribution of correct answers per participant.

In the demonstration experiment, the average freshness value measured by the Fish analyzer was 0.341, with a standard deviation of 0.072 (The scale range used by the Fish analyzer is 0–1. A value above 0.43 indicates that the fish is fresh enough to be eaten raw. A value of 0.341, as mentioned in the manuscript, is considered moderately fresh, meaning the fish is recommended to be cooked rather than eaten raw). Seven participants did not provide any correct answers in the ten quizzes, while four of the highest-scoring participants recorded eight correct answers. For these four participants, the maximum difference in freshness values between incorrectly selected fish and the freshest fish was 0.093, with the average being 0.047. The average number of correct answers was 3.78, and the standard deviation was 0.49. This average was higher than 3.33 (the expected value of the number of correct answers hypothesizing a binomial distribution if the answer was randomly selected). Figure 3 shows the number of participants for each number of correct answers with the expected number of participants presumed in the binomial distribution [35]. We conducted a chi-square test using the number of participants for each number of correct answers compared with the expected value of the number of participants for each number of correct answers [36]. The sum of the chi-square value was 53.48, and the shape of the distribution was statistically different from the random selection (critical value of α = 0.005 is 25.2).

### 3.2. Correlation

Table 3, Table 4 and Table 5 present the correlation matrices for the consumer judgments, actual measured freshness, and visual cues.

Gloss, length, PC1_C, PC3_C, PC6_C, and PC8_S are positively correlated with actual measured freshness. This means that higher values of these variables indicate higher actual freshness of the fish. Eye luminance (eye_lum), PC7_C, PC2_S, PC5_S, and PC6_S are negatively correlated with freshness, indicating that higher values of these variables correspond to lower actual freshness of the fish.

The correlation between the consumer judgments and the freshness measured by the Fish analyzer is only 0.08 and not significant. Consumer judgments are significantly and positively correlated with fish eye luminance (eye_lum) and PC1_C. This means that the brighter the fish’s eyes or the higher the PC1_C value, the fresher the consumers perceive the fish to be. Conversely, consumer judgments are significantly negatively correlated with PC2_C and PC3_C. This indicates that higher values of these principal components lead consumers to believe the fish is less fresh. There are no statistically significant correlations between the measured data and shape data with consumer judgments.

### 3.3. Participant Grouping and ANOVA

To investigate the differences in freshness judgment preferences between participants with high and low correctness rates, we categorized the participants based on their accuracy. We standardized the information on the characteristics of the fish selected by each participant to show their preference when judging freshness. The participants in each group were as follows: Group L (low correctness rate): individuals with zero or one correct answers; Group M (average correctness rate): individuals with four correct answers; and Group H (high correctness rate): individuals with seven or eight correct answers. These groups contained 39, 141, and 20 respondents, respectively. We compared the average scores of each group and performed an analysis of variance.

**Figure 4:**
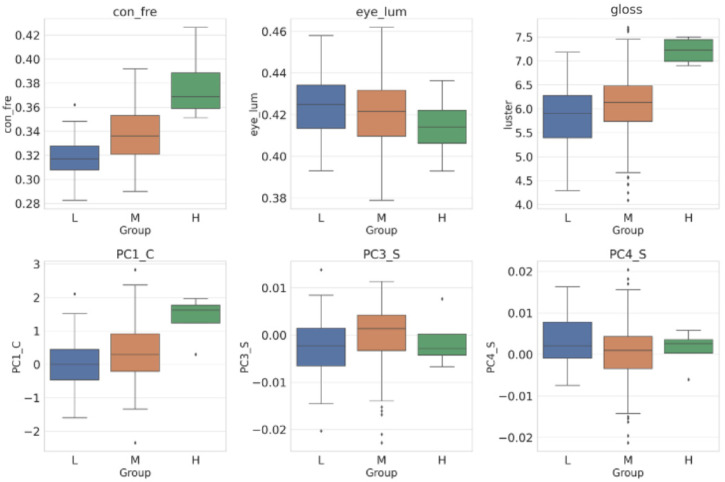
Boxplots of significant variables at 95% confidence intervals. Note: con_fre: measures freshness. Eye_lum represents eye luminance. PC1_C: As the PC1 value increases, the colors in parts P1, P3, P5, and P6 become more yellow. PC3_S: Reflects the size difference in the caudal fin relative to the body. Changes in PC3 values indicate the size difference between the caudal fin and the body. PC4_S: Reflects the thickness of the caudal peduncle relative to the body. Changes in PC4 values indicate differences in the thickness of the caudal peduncle and body size.

Figure 4 highlights the variables that exhibited significant differences among the low, medium, and high correctness rates (LMH) groups (*p* < 0.05). Notably, these groups showed significant variations in measured freshness (con_fre), gloss, color characteristics (PC1_C), and body shape features (PC3_S and PC4_S).

Significant differences were observed in preference for eye luminance between the low and high correctness rate (LH) groups. The participants in Group H predominantly selected fish with lower eye luminance, whereas those in Group L were inclined towards fish with higher luminance. When it came to gloss, Group H generally preferred fish with high gloss, while Group L opted more for fish with low gloss. Regarding PC1_C, Group H favored photos with a higher PC1_C component, indicating a preference for certain color traits, whereas Group L selected photos with a lower PC1_C component. For the body shape, there were differences in the choices of Groups L and H concerning the body shape characteristics PC3_S and PC4_S.

## 4. Discussion

The primary aim of this study is to investigate whether and how consumers can judge the freshness of fish sold in the real market, but not to assess the general ability of consumers to determine freshness in a controlled environment where fish originating from a single location can be used. Thus, we focus on fish sold in real-world settings. In practice, fish sold in the market can vary significantly due to factors such as growth stages, environmental conditions of the habitat, and treatments after the harvests, which substantially impact appearance and shape. These variations may be more significant than those caused by differences in freshness alone. Consumers must therefore discern freshness amid these factors.

Principal component analysis (PCA) was chosen for this study because it allows for a broad extraction of changes without forcing variations into dominant factors, which happens in factor analysis. When conducting factor analysis with a controlled sample, factors related to freshness would naturally emerge as the most significant. However, with market samples, other variables could dominate, potentially overshadowing the visual changes caused by freshness. PCA, as merely a diagonalization of the variance–covariance matrix, prevents minor factors from being excluded in favor of dominant ones. This flexibility is essential for our study, where we aim to capture consumers’ more subtle visual cues to assess the freshness of fish sold in real-world settings.

The results demonstrate that while the first principal component of color was correlated with freshness, other components were not. It was also found that most factors related to shape showed no significant correlation with freshness. These results were anticipated, as freshness changes are more likely to appear in color than shape, which is more influenced by environmental and growth factors. This supports the validity of using PCA in this analysis.

### 4.1. Accuracy of Visual Judgment of Freshness of Participants

The correlation coefficient between the measured freshness and frequency of selection by participants was 0.08, which is very low and statistically not significant, though it is positive. This result does not provide evidence that common people can accurately judge the freshness of fish using visual cues alone. Previous studies have shown that multiple sensory factors—visual, olfactory, and tactile cues—are typically used to assess freshness [15,37], and the low correlation observed in this study (r = 0.08) supports the idea that many consumers rely on sensory information beyond just visual cues, such as smell and touch. However, our study has revealed that a small number of consumers can accurately assess freshness based on visual cues alone. The chi-square analysis between our choice experiment result and random selection showed that the accuracy of judgment was higher than that of random selection. This means that there are participants who can accurately judge the freshness. For instance, four participants correctly judged the freshness in eight out of ten choice quizzes, suggesting that some individuals possess a higher capability to make accurate freshness judgments, even when relying only on visual information.

For the binomial distribution, the accuracy of the visual judgment of the freshness of participants was 3.78 (SD = 0.49). From this, we estimate that 37.8% of Japanese individuals gave correct answers, which is higher than the expected value, assuming a binomial distribution. However, the capacity for freshness estimation varies widely from one person to another. This is depicted in Figure 3, where seven participants could not provide even one correct answer. Conversely, four other participants achieved eight correct answers (and 80% depicts extremely high judgment accuracy when we consider the existence of similar fresh fish in alternatives of selection quizzes). The observed value line is on the right side of the expected value line in Figure 3. From this, we can conjecture that there were participants who could detect freshness from photographs of fish with high accuracy, although the sensitivity and accuracy differed among general consumers.

### 4.2. Correlations

As the result of correlation analysis, two characteristics of fish and four principal components were positively correlated with measured freshness, namely gloss (0.30), length (0.25), PC1_C (0.51), PC3_C (0.23), PC6_C (0.15), and PC8_S (0.14), and one characteristic of fish and four principle components were negatively correlated with measured freshness, eye luminance (−0.17), PC7_C (−0.17), PC2_S (−0.20), PC5_S (−0.26), and PC6_S (−0.19). Among these, the highest absolute value of the correlation coefficient was 0.51 of PC1_C. Among these features and principal components, there is eye luminance (0.59), PC1-C (0.17), and PC3-C (−0.18) which are correlated with selection frequency. In addition to this, PC2_C (0.38) was positively correlated with selection frequency, but the correlation with measured freshness was small. As a whole, the correlation coefficients were low and there were no characteristic and principal components that could be used as a proxy for freshness in the fish analyzer. Therefore, we estimated that participants who were able to select the fish photos with the highest freshness would use a combination of this visual information. Based on the hypothesis of the combined use of visual information from photographs, we will discuss below how participants utilized the visual information.

Before accepting the hypothesis that common consumers can detect freshness only from the visual information in photographs of fish, we must demonstrate the possible mechanism of freshness detection by general consumers. The correlation analysis showed that there were 12 possible components for the detection of freshness, namely gloss, length, eye luminance, PC1_C, PC2_C, PC3_C, PC6_C, PC7_C, PC2_S, PC5_S, PC6_S, and PC8_S. Body size can be detected by comparing body length to the size of the dish under the fish’s body. Theoretically, the relationship between body size and freshness should be considered [38]. In principle, body size is not directly related to freshness. This correlation may be caused by differences in treatment during the distribution process because large fish are sold at high prices and treated carefully [39]. Therefore, we considered the correlation between measured freshness and length as a spurious correlation and removed length from the candidates of possible components.

Gloss is also obtained directly from the fish. It is the ratio of positive reflection to diffuse reflection and is measured as an index of the smoothness and glitter of the body’s surface [40]. Gloss cannot be measured directly from photographs; however, humans can detect the smoothness and glitter of a surface from photographs and can estimate the gloss of the fish. We need to clarify this possibility in future studies. Therefore, we used gloss as a candidate mechanism.

The frequency of selection refers to the participants’ general preference for fresh fish. As presented in Table 3, only 4 components were frequently used to judge freshness by the participants; PC6_C, PC7_C, PC2_S, PC5_S, PC6_S, and PC8_S, which have a relatively high correlation with freshness, were not widely used as freshness indicators. One possible reason for the limited use of these components is that most participants ignored these signals because they did not have the knowledge. Another possible reason is that the sensitivities of detecting these signals differ between machines and humans, and humans cannot commonly detect the difference in these signals. When we accept this hypothesis, there remains a possibility that several gifted human beings can detect the difference with high sensitivity. The experiment in this study was not designed for the evaluation of sensitivities of gifted persons. We need future studies to clarify the content and mechanism of their gifted ability and the possibility of education to improve the ability for common people.

Another notable finding from the correlation analysis is that consumers’ knowledge is not always accurate. Most participants gave negative evaluations of PC2_C, but the scores of this component were not correlated with measured freshness. Additionally, there were typical misinterpretations in the correlations between consumer judgments and eye luminance, as well as PC3_C. Freshness is inversely correlated with eye luminance (−0.17) and positively correlated with PC3_C (0.28). However, selection scores are positively correlated with eye luminance (0.59) and negatively correlated with PC2_C (−0.18). This indicates that many participants may perform poorly due to misinterpretations of the visual information in the photographs.

**Figure 5:**
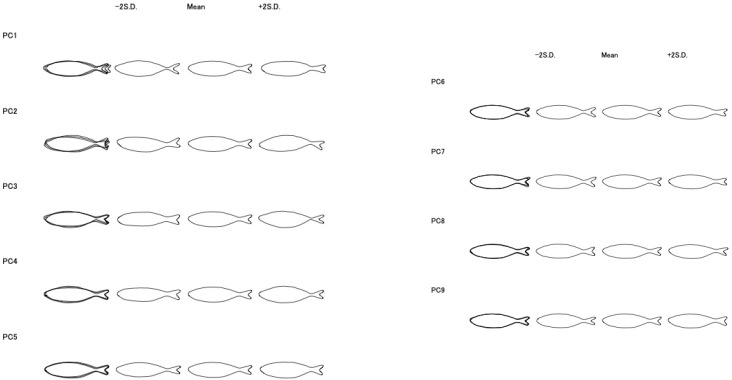
Morphological changes in body shape to changes in principle component score. Note: −2 SD: number of imaginary fish for which the principle component score is at the value of mean − 2 standard deviations; mean: number of imaginary fish for which the principle component score is at mean; +2 SD: number of the imaginary fish for which the principal component score is at the value of mean + 2 standard deviations.

Figure 5 shows the morphological changes with changes in each principal component score by the contour of −2 SD, mean and +2 SD imaginary individuals.

People do not place much emphasis on shape information when judging freshness. PC2_S and PC5_S are inversely correlated with freshness. In Figure 5, the ratio of body width to length is high in +2 SD imaginary fish compared to −2 SD and the mean imaginary fish of PC5_S. This indicated that PC5_S represented obesity. This is possibly due to obese fish being more prone to damage during transportation or handling. The changes in PC5_S revealed a potential relationship between the fish’s body shape and how it was handled in the market distribution process. The meanings of PC6_S and PC8_S were difficult to recognize from only the contours of the fish. When we observed differences in the shape of the caudal fin, the contours also differed. Fins are easily damaged during rough treatments. These differences may have occurred in the process of transportation or management of shops. Fin vulnerability may be related to freshness, and physiological and biochemical changes after death can cause morphological changes in caudal fins. Clarifying the mechanism of the changes in the shape of the caudal fin is a future challenge.

### 4.3. Participant Grouping and ANOVA

As described in Section 3.3, participants were categorized based on their accuracy in judging fish freshness. Participants who provided seven or eight correct answers were classified into the “High-accuracy (H)” group, which comprised 20 individuals. The probability of randomly selecting seven or more correct answers without relying on visual freshness information is 0.020. Given the sample size of 529 respondents, the expected number of participants with such accuracy is 10.40. A chi-square test showed that the probability of 20 participants having seven or more correct answers is 0.0026, indicating that nearly half of these participants accurately judged freshness based on visual information. This group was categorized as the “High-accuracy (H) group.

For the “Medium-accuracy (M)” group, participants with four correct answers were selected. A correct answer count of fouris the mode of the data and includes the mean of 3.7. The probability of obtaining four correct answers by random selection is 0.228, and given 529 respondents, the expected number of participants with four correct answers would be 120, while the actual number was 131, which is close to the expected value. This suggests that this group is mainly consistent with random selection and that participants in this group likely did not have a clear visual standard for assessing freshness or could not detect relevant visual information. We categorized this group as the “Medium-accuracy (M) group”.

Lastly, participants with zero or one correct answers were categorized into the “Low-accuracy (L)” group, comprising 39 individuals. In such surveys, it is possible that some participants may have intentionally chosen incorrect answers, while others might have detected changes in color and shape associated with freshness but misinterpreted these cues, leading to inaccurate judgments. If participants chose answers randomly because they could not visually assess freshness, the probability of achieving a correct answer count of one or lower is 0.104. Given 529 respondents, the expected number of individuals with this level of performance would be 55.5. However, the observed number was lower, at 39, suggesting that few participants either deliberately avoided correct answers or misunderstood the visual cues they detected. Although the possibility of misinterpretation cannot be entirely ruled out, these participants were classified as the “Low-accuracy (L)” group and were included in the subsequent ANOVA analysis.

As depicted in Figure 4, Group H preferred to choose fish with lower eye luminance, whereas Group L tended to select fish with higher luminance. From the previous results, we know that the luminance of fish’s eyes is inversely related to fish freshness, which means that the participants in Group H made correct choices using fish eye luminance. Most participants in Group L made incorrect choices using eye luminance information. This may be part of the reason for the difference in correctness.

Regarding the choice of gloss, Group H tended to select photos of fish with high gloss, whereas Group L tended to select pictures of fish with low gloss. From this, we hypothesize that individuals with higher accuracy can recognize the difference in gloss on the surface of fish more efficiently through photographs. This implies that the gloss on a fish’s surface may be an important visual cue that influences an individual’s judgment of fish quality. Regarding PC1_C (color characteristics), Group H tended to select photos with a high PC1_C component, whereas Group L selected pictures with a lower component. The high correlation between PC1_C and the measured freshness suggests that people in Group H could correctly determine the freshness of fish by looking at the color profile of PC1_C. This might be an essential factor that contributed to the difference in accuracy between groups.

There were differences in the choices of Groups L and H concerning the body shape characteristics PC3_S and PC4_S. However, the differences in the mean values were small and the correlations with freshness were weak. To some extent, these signs might have misled their judgment.

## 5. Conclusions

This study demonstrates that several Japanese consumers evaluate fish freshness based on visual attributes, body color, and eye luminance, although assessment accuracy varies among consumers. Building on previous research by Nakamura et al. [11] and Murakoshi et al. [17], which demonstrated that professionals could judge freshness based on appearance and eye luminance, our findings indicate that differences in consumer accuracy were partially due to low-accuracy consumers misinterpreting visual signs.

Jarmin et al. [12] and Rocculi et al. [13] found that color changes in fish eyes and gills correlated with fish deterioration. Our study built upon this by including multiple types of visual information (i.e., color and shape) in our analysis, allowing us to shed more light on the consumer freshness assessment process. The accurate participants of the experiment selected fish for the colors in parts P1, P3, P5, and P6, and more yellow and bright colors in the abdominal region. This suggested that the difference in body color could be due to changes in chromatophores caused by nerve control after the fish’s death. These findings align with the observations of [41] regarding the importance of visual factors in food choices.

Our study also compares data from fish analyzers with consumer judgment, as [20] pioneered. This approach allows us to delve into the nuances of consumer sensitivity to specific visual signals. Combining objective measurements from a fish analyzer with consumer assessments helps us better understand the complexity of visual cues in fish freshness detection.

In summary, Japanese consumers evaluate fish freshness based on visual attributes, including body color, eye luminance, etc., even though the judgments are not always correct. The critical role of visual factors in consumer decision-making but is often ignored in product presentation and marketing strategies in the seafood industry. Future research can further explore the cognitive and cultural factors influencing these visual preferences and investigate the physiological and biochemical aspects of chromatophores to deepen our understanding of the complexities behind these visual cues.

## Figures and Tables

**Table 1:** Description of Color Principal Component.

Principal Component	Description
PC1_C	As the PC1 value increases, the colors in parts P1, P3, P5, and P6 become more yellow.
PC2_C	As the PC2 value increases, the luminance in part P1 decreases, while the redness in part P3 increases.
PC3_C	As the PC3 value increases, the green in part P2 increases, along with its luminance.
PC4_C	As the PC4 value increases, the luminance in parts P2 and P3 increases, while parts P1 and P6 shift towards red.
PC5_C	As the PC5 value increases, the luminance in parts P1 and P6 increases.
PC6_C	As the PC6 value increases, the luminance in part P4 increases, with a shift towards blue.
PC7_C	As the PC7 value increases, the luminance in part P5 increases, and part P2 shifts towards blue.
PC8_C	As the PC8 value increases, the luminance in part P6 increases, and part P4 shifts towards yellow.
PC9_C	As the PC9 value increases, the luminance in part P4 increases, the redness in parts P1 and P4 increases, and the greenness in parts P2 and P6 increases.
PC10_C	As the PC10 value increases, the redness in part P3 increases.

Note: Descriptions were made from principal component loadings of L, a, and b values in each part in the first step. The validity of these descriptions was confirmed by comparing the highest and lowest photographs of fish in each principal component in the second step.

**Table 2:** Description of shape principal component.

Principal Component	Description
PC1_S	Reflects the size and spread of the caudal fin relative to the body. Lower PC1 values indicate a larger and more spread-out caudal fin.
PC2_S	Reflects the orientation of the caudal fin relative to the body axis. Changes in PC2 values are associated with the direction of the caudal fin.
PC3_S	Reflects the size difference in the caudal fin relative to the body. Changes in PC3 values indicate the size difference between the caudal fin and the body.
PC4_S	Reflects the thickness of the caudal peduncle relative to the body. Changes in PC4 values indicate differences in the thickness of the caudal peduncle and body size.
PC5_S	Reflects the ratio of overall length to body height. Changes in PC5 values indicate the proportion difference between total body length and body height.
PC6_S	Reflects the variation in the spread of the caudal fin. Changes in PC6 values indicate different ways the caudal fin spreads.
PC7_S, PC8_S, PC9_S	Mainly reflect the shape of the caudal fin. PC8, in particular, shows the feature of the upper part of the caudal fin s extending upwards.

Note: The descriptions were made by comparing three contours provided by Shape: average +2 standard deviations, average, and average −2 standard deviations for each principal component in the first step. Then, the validity of the descriptions was confirmed by comparing the highest and lowest photographs of fish in each principal component in the second step.

**Table 3:** Correlation matrix for measured data.

	Score	Con_Fre
Con_fre	0.8	
gloss	0.05	0.3 **
lipid contents	0.08	−0.12
length	0.05	0.25 **
weight	0.06	0.1

Notes: ** Correlation is significant at the 0.01 level, correlation is significant at the 0.05 level. con_fre: measured freshness.

**Table 4:** Correlation matrix for eye luminance and color data.

	Score	Con_Fre
eye_lum	0.59 **	−0.17 *
PC1_C	0.17 *	0.51 **
PC2_C	−0.36 **	0.1
PC3_C	−0.18 **	0.23 **
PC4_C	0.02	0.01
PC5_C	−0.07	0.03
PC6_C	0.11	0.15 *
PC7_C	0.04	−0.17 *
PC8_C	0.02	−0.04
PC9_C	−0.02	0.06

Notes: ** Correlation is significant at the 0.01 level, * Correlation is significant at the 0.05 level. con_fre: measured freshness. For the meaning of color principal components, please refer to Table 1.

**Table 5:** Correlation matrix for shape data.

	Score	Con_Fre
PC1_S	−0.1	−0.05
PC2_S	−0.04	−0.2 **
PC3_S	0.01	−0.03
PC4_S	0.13	−0.12
PC5_S	−0.01	−0.26 **
PC6_S	−0.09	−0.19 **
PC7_S	−0.06	−0.02
PC8_S	0	0.14 *
PC9_S	0.02	0.04

Notes: ** Correlation is significant at the 0.01 level, * Correlation is significant at the 0.05 level. con_fre: measured freshness. For the meaning of shape principal components, please refer to Table 1.

## Data Availability

The data that support the findings of this study are available from the corresponding author upon reasonable request.

## References

[1] Ali J., Kapoor S., Moorthy J. (2010). Buying behaviour of consumers for food products in an emerging economy. Br. Food J..

[2] Sakai Y., Tada T., Nomura T., Yagi N. (2024). The Welfare Value of Freshness: A Hedonic Price Analysis in the Retail Seafood Market in Japan. Mar. Resour. Econ..

[3] Brunsø K., Verbeke W., Ottar Olsen S., Fruensgaard Jeppesen L. (2009). Motives, barriers and quality evaluation in fish consumption situations. Br. Food J..

[4] Benn Y., Webb T.L., Chang B.P., Reidy J. (2015). What information do consumers consider, and how do they look for it, when shopping for groceries online?. Appetite.

[5] Sakai Y., Kurokura H., Tada T., Nomura T., Yagi N. (2023). Quality distribution of fresh fish and retailers’ sales strategies in special wards of Tokyo. Fish. Sci..

[6] Cheng J., Sun D., Zeng X., Liu D. (2015). Recent advances in methods and techniques for freshness quality determination and evaluation of fish and fish fillets: A review. Crit. Rev. Food Sci. Nutr..

[7] Madhubhashini M.N., Liyanage C.P., Alahakoon A.U., Liyanage R.P. (2024). Development of a comprehensive classification model for determining the storage day of frigate tuna (Auxis thazard) for freshness evaluation using a portable electronic nose. Int. J. Food Sci. Technol..

[8] Hassoun A., Karoui R. (2017). Quality evaluation of fish and other seafood by traditional and nondestructive instrumental methods: Advantages and limitations. Crit. Rev. Food Sci. Nutr..

[9] Prabhakar P.K., Vatsa S., Srivastav P.P., Pathak S.S. (2020). A comprehensive review on freshness of fish and assessment: Analytical methods and recent innovations. Food Res. Int..

[10] Yuan P., Wang Y., Miyazaki R., Liang J., Hirasaka K., Tachibana K., Taniyama S. (2018). A convenient and nondestructive method using bio-impedance analysis to determine fish freshness during ice storage. Fish. Sci..

[11] Nakamura M., Matsumoto K., Morimoto E., Ezoe S., Maeda T., Hirano T. (2011). Model of auctioneer estimation of swordtip squid (Loligo edulis) quality. Kansei Eng. Int. J..

[12] Jarmin R., Khuan L.Y., Hashim H., Rahman N.H.A. A comparison on fish freshness determination method. Proceedings of the Paper Presented at the 2012 International Conference on System Engineering and Technology (ICSET).

[13] Rocculi P., Cevoli C., Tappi S., Genovese J., Urbinati E., Picone G., Fabbri A., Capozzi F., Dalla Rosa M. (2019). Freshness assessment of European hake (Merluccius merluccius) through the evaluation of eye chromatic and morphological characteristics. Food Res. Int..

[14] Karoui R., Lefur B., Grondin C., Thomas E., Demeulemester C., Baerdemaeker J.D., Guillard A. (2007). Mid-infrared spectroscopy as a new tool for the evaluation of fish freshness. Int. J. Food Sci. Technol..

[15] Olafsdottir G., Nesvadba P., Di Natale C., Careche M., Oehlenschläger J., Tryggvadóttir S.V., Schubring R., Kroeger M., Heia K., Esaiassen M. (2004). Multisensor for Fish Quality Determination.

[16] Hicks D., Pivarnik L., McDermott R. (2008). Consumer perceptions about seafood—An Internet survey. J. Foodserv..

[17] Murakoshi T., Masuda T., Utsumi K., Tsubota K., Wada Y. (2013). Glossiness and Perishable Food Quality: Visual Freshness Judgment of Fish Eyes Based on Luminance Distribution. PLoS ONE.

[18] Costa C., Antonucci F., Menesatti P., Pallottino F., Boglione C., Cataudella S. (2013). An Advanced Colour Calibration Method for Fish Freshness Assessment: A Comparison Between Standard and Passive Refrigeration Modalities. Food Bioprocess Technol..

[19] Ohta K. (2004). Treatment for asthma with biological agents. Jpn. J. Clin. Immunol..

[20] Sakai Y., Nakamura A., Yagi N., Suzuki T., Oishi T., Kurokura H. (2018). Consumers’ attitude toward inspection methods and institutions for potential radioactive contamination: A choice-based conjoint analysis. J. Int. Fish..

[21] Botta J.R. (1996). Evaluation of Seafood Freshness Quality.

[22] Yamato Scale Co., Ltd. Fish Analyzer Operation Manual; Assurant Innovations. https://assurantinnovations.com/wp-content/uploads/2021/10/Fish-Analyzer-Operation-Manual.pdf.

[23] Ishihara S., Taketani B., Ioka H., Kiyokawa T. (2018). Nondestructive Determination of Freshness of Roundnose Flounder by Impedance Analysis. Bull. Shimane Prefect. Fish. Technol. Res. Cent..

[24] Fan X., Lin X., Wu C., Zhang N., Cheng Q., Qi H., Konno K., Dong X. (2021). Estimating freshness of ice storage rainbow trout using bioelectrical impedance analysis. Food Sci. Nutr..

[25] Okabe S., Shoichi M. (2019). Technical Explanation and Application Examples of Fish Analyzer. Teichi.

[26] Koseki S., Kitakami S., Kato N., Arai K. (2006). Rigor mortis of fish and shellfish and evaluation of freshness of their muscles as K value. J. Sch. Mar. Sci. Technol. Tokai Univ..

[27] Ministry of Agriculture, Forestry and Fisheries (MAFF) Efforts Based on the Basic Plan for the Promotion of Food Education. https://www.maff.go.jp/chushi/syokuiku/katudou/torikumi/attach/pdf/r02-6.pdf.

[28] Wang F., Zang Y., Wo Q., Zou C., Wang N., Wang X., Li D. Fish freshness rapid detection based on fish-eye image. Proceedings of the Paper Presented at the PIAGENG 2013: Image Processing and Photonics for Agricultural Engineering 2013.

[29] Iwata H., Ukai Y. (2002). SHAPE: A computer program package for quantitative evaluation of biological shapes based on elliptic Fourier descriptors. J. Hered..

[30] Bro R., Smilde A.K. (2014). Principal component analysis. Anal. Methods.

[31] Shlens J. (2014). A tutorial on principal component analysis. arXiv.

[32] Wu T., Yang L., Zhou J., Lai D.C., Zhong N. (2019). An improved nondestructive measurement method for salmon freshness based on spectral and image information fusion. Comput. Electron. Agric..

[33] Weatherall I.L., Coombs B.D. (1992). Skin color measurements in terms of CIELAB color space values. J. Investig. Dermatol..

[34] St L., Wold S. (1989). Analysis of variance (ANOVA). Chemom. Intell. Lab. Syst..

[35] Agresti A. (2012). Categorical Data Analysis.

[36] McHugh M.L. (2013). The chi-square test of independence. Biochem. Medica.

[37] Gvili Y., Tal A., Amar M., Wansink B. (2017). Moving up in taste: Enhanced projected taste and freshness of moving food products. Psychol. Mark..

[38] Makowski D., Ben-Shachar M.S., Patil I., Lüdecke D. (2020). Methods and algorithms for correlation analysis in R. J. Open Source Softw..

[39] Hirose A., Yoshitake M., Onodera J., Ooba K., Sakakibara T., Ito A., Ashida S., Shiina Y. (2016). Measurement of fat content of cultured Pacific bluefin tuna Thunnus orientalis by near-infrared spectroscopy. Nippon. Suisan Gakkaishi.

[40] Hua L., Shen J., Chen Y., Lan Q., Liu J. (2020). Wipe-on and durable self-cleaning coating for glass facade. Thin Solid Film..

[41] Vermeir I. (2020). How Visuals Affect Food Choice. Foods.

